# Knowledge of contraceptive effectiveness and method use among women in Hanoi, Vietnam

**DOI:** 10.1016/j.conx.2019.100009

**Published:** 2019-07-11

**Authors:** Maria F. Gallo, Nghia Nguyen, Chuong Nguyen, Markus J. Steiner

**Affiliations:** a The Ohio State University, College of Public Health, Division of Epidemiology, Cunz Hall, 1841 Neil Avenue, Columbus, OH, 43210, USA; b Department of Obstetrics and Gynecology, Vinmec International Hospital, 458 Minh Khai, Hanoi, Vietnam; c Department of Research and Training, Hanoi Obstetrics and Gynecology Hospital, La Thanh Road, Hanoi, Vietnam; d Contraceptive Technology Innovation Division, FHI 360, 359 Blackwell Street, Durham, NC 27701, USA

**Keywords:** Contraception, Education, Knowledge, Women, Vietnam

## Abstract

**Objective:**

To evaluate the association between contraceptive knowledge and type of method used.

**Methods:**

We analyzed data from a cross-sectional study of sexually active women in Hanoi, Vietnam, not desiring pregnancy. We used linear and logistic regression to evaluate contraceptive knowledge of users of the intrauterine device (IUD), combination oral contraception (COC) and male condoms. We measured contraceptive knowledge with seven questions on relative effectiveness of methods, reversibility, covert use, contraindications and side effects.

**Results:**

Respondents used IUD (*n* = 128), COC (*n* = 126) or condoms (*n* = 167). Summary knowledge scores did not differ by current type of method used. Only one knowledge domain, contraceptive effectiveness, varied by method. Compared to condom users, IUD users had higher odds of correctly identifying the IUD as more effective than COC, condoms and withdrawal (adjusted odds ratio [aOR], 4.8; 95% confidence interval [CI], 2.7–8.3). Higher proportions of condom users (49.7%) mistakenly identified condoms as the most effective of listed methods compared to IUD (20.3%) and COC users (23.0%). On the other hand, IUD and COC users had lower odds (aOR, 0.5; 95% CI, 0.2–1.0 and aOR, 0.3; 95% CI, 0.1–0.6, respectively) of identifying consistent condom use as better for pregnancy prevention than other practices (e.g., withdrawal and postcoital douching).

**Conclusions:**

IUD users more often recognized that the IUD is highly effective while condom users appeared to overestimate condom effectiveness. Contraceptive counseling should ensure that women understand the relative effectiveness of methods. We found no evidence that other types of contraceptive knowledge differed by type of method used.

**Implications:**

Knowledge of contraceptive effectiveness was the sole difference detected in contraceptive knowledge between women in Hanoi, Vietnam, using the IUD, COC or male condoms.

## 1 Introduction

Poor knowledge about contraception has been documented in a variety of settings worldwide [1], [2], [3]. This lack of knowledge has been identified as a possible determinant of failure to consistently and correctly use effective contraception and, subsequently, as a key cause of unintended pregnancy [4], [5]. Women themselves have recognized the importance of their lack of knowledge. For example, female university students attributed their failure to use long-acting reversible contraception (LARC) to their lack of knowledge of the methods [6], and women in a postnatal ward cited their lack of knowledge of postpartum methods [7].

Numerous interventions have been developed to improve contraceptive knowledge [8]; these are predicated on the assumption that a certain degree or type of knowledge is needed to prompt behavior change related to contraception use. For example, using the transtheoretical model as a framework [9], we can view having contraception-related knowledge as a required component for women to transition from precontemplation stage to contemplation stage. The precontemplation stage could be characterized as including those who are not even considering the use of contraception, while the contemplation stage could include those who recognize that contraception use is needed to effectively prevent pregnancy. This progression is necessary for eventually undertaking actions related to initiating and maintaining the use of contraception.

However, despite demonstrating success in improving contraceptive knowledge, few educational interventions have been shown to influence the behavioral change of initiating or continuing use of contraception or prevention of pregnancy [8]. Education alone might be insufficient for overcoming other, possibly more salient barriers to contraception use (e.g., geographic or financial access, concerns about side effects, partner-related dynamics, and religious or sociocultural norms). Alternatively, educational interventions might fail to change behavior if they do not improve women's knowledge of the specific information needed to successfully adopt and continue use of contraception. Recently, a 25-item Contraceptive Assessment Tool was developed in New York City as a standardized measure of knowledge for clinical practice and research [10]. The importance of the individual items on the tool, though, is unknown.

Understanding the effect of knowledge on contraception use is critical for informing interventions to prevent unintended pregnancy. We evaluated the association between contraceptive knowledge and use of contraception among sexually active, reproductive-age women in Hanoi, Vietnam, who did not desire pregnancy. Our objective was to determine the degree to which having accurate contraceptive knowledge (overall level and specific types of knowledge) was associated with current use of the intrauterine device (IUD), combination oral contraception (COC) or male condoms.

## 2 Materials and methods

We conducted a secondary analysis of data from a cross-sectional study of 500 adult women of reproductive age (18–45 years) attending the obstetrics-gynecology department of a large public hospital in Hanoi, Vietnam, for routine care in 2017–2018. The primary study objective of this cross-sectional study was to evaluate a novel method for measuring women's implicit beliefs about contraceptive safety; these findings will be reported elsewhere. To be eligible to participate, women had to have at least a minimal level of literacy, report being comfortable using a computer, be sexually active (defined as ≥ 1 penile–vaginal act in past month), not be pregnant or breastfeeding, and not want a pregnancy within the next 12 months. Women provided written consent before enrollment, and The Ohio State Biomedical Sciences Institutional Review Board (IRB) and the Hanoi School of Public Health IRB approved the research.

Vietnam's family planning program historically has promoted and provided the copper IUD, with a limited number of other methods (i.e., COC, female sterilization and male condoms) introduced only more recently during the economic reforms of the late 20th century [11]. In contrast, use of other modern methods (e.g., implants, injectables, patches and diaphragms) is rare. Thus, the sample was stratified to enroll three subgroups: (1) women who were not currently using nor were initiating use during the study visit of the IUD or COC, (2) IUD users and 3) COC users. For this secondary analysis, we restricted the analysis population to those who were currently using the IUD, COC or male condoms.

We administered seven questions on respondent knowledge of contraception adapted from the Contraceptive Assessment Tool [10]. Questions were developed to measure attributes of contraceptive methods that we theorized could be important for women to understand before deciding to initiate use of the IUD or COC. These components included the relative effectiveness of methods, reversibility, ability to use covertly, contraindications and incorrect side effects. Study staff read the possible response options for each question and asked women to select the best single response. Women also were surveyed on their demographics and sexual and reproductive-health history and behaviors. We used REDCap electronic data capture tool [12] for collecting and managing study data.

We created a contraceptive knowledge score by summing the correct number of responses for the contraceptive knowledge questions (possible score range, 0–7). We used linear regression to evaluate the association between current use of IUD or COC (i.e., a dichotomous variable for using either method vs. neither) and the summary knowledge score. We also fit seven logistic regression models to evaluate each contraceptive knowledge question individually. We then fit multivariable linear and logistic regression models to adjust for the following possible confounders of the association between IUD/COC use and contraceptive knowledge based on the literature [1], [2], [3], [4], [5], [6]: age (continuous variable), residence (city vs. town or rural area), education (secondary or lower vs. higher) and monthly household income (< 15 million Vietnamese dong [equivalent to ~ 650 US dollars] vs. higher). We selected 15 million dong as the threshold as this is often used by the Vietnamese government for identifying high-income households. We intended to adjust also for marital status and Kinh ethnicity; however, these variables did not have sufficient variation to include in the modeling. We used SAS 9.4 (SAS, Cary, NC, USA) for all analyses.

## 3 Results

Among the women recruited and then screened for participation, 508 women were eligible for the study. Eight women declined, and thus, 500 women were enrolled. We restricted the present analysis to the 421 respondents who reported current use of the IUD (*n* = 128), COC (*n* = 126) or male condoms only (*n* = 167). The mean age was 33.9 years (standard deviation [SD], 5.3). Most women resided in a city (88.3%–94.4%), were married (93.4%–100.0%) and were of Kinh ethnicity (94.0–97.6%) (Table 1).

**Table 1:** Demographics and other characteristics and behaviors by contraceptive method use among sexually active women not desiring pregnancy in Hanoi, Vietnam, in 2017–2018

	IUD (*n* = 128)		COC (*n* = 126)		Male condoms (*n* = 167)	
	*n*	(%)	*n*	(%)	*n*	(%)
Age in years						
21–31	30	(23.4)	38	(30.2)	70	(41.9)
32–36	48	(37.5)	45	(35.7)	49	(29.3)
37–45	50	(39.1)	43	(34.1)	48	(28.7)
Residence						
City	113	(88.3)	119	(94.4)	152	(91.0)
Town or rural area	15	(11.7)	7	(5.6)	15	(9.0)
Marital status						
Never married	0	(0.0)	1	(0.8)	10	(6.0)
Married	128	(100.0)	124	(99.2)	156	(93.4)
Separated, divorced, widowed	0	(0.0)	0	(0.0)	1	(0.6)
Ethnicity						
Kinh	121	(94.5)	123	(97.6)	157	(94.0)
Non-Kinh	7	(5.5)	3	(2.4)	10	(6.0)
Highest level of education completed						
Primary or lower secondary	15	(11.7)	5	(4.0)	5	(3.0)
Upper secondary	28	(21.9)	15	(11.9)	34	(20.5)
Higher	85	(66.4)	106	(84.1)	127	(76.5)
Monthly household income						
Less than 15,000,000 Vietnamese dong	29	(25.9)	24	(21.1)	23	(15.5)
At least 15,000,000 Vietnamese dong	83	(74.1)	90	(78.9)	125	(84.5)

Only 30.6% of women correctly identified the IUD as the most effective method for preventing pregnancy from the response options presented (i.e., the pill, male condom, withdrawal or “pull-out” or all equally effective), and only 19.0% of women accurately answered that the IUD can be used by women of all ages, used by nulligravid women, and inserted immediately after birth or having an abortion (Table in Appendix). Finally, only 58.7% of women recognized that the contraceptive pill, shot and implant do not cause infertility. The remaining knowledge questions — which involved the effectiveness of condoms for pregnancy and disease prevention, method reversibility and ability to use covertly — were answered correctly by greater proportions of women.

Respondents had a mean summary knowledge score of 4.6 (SD, 1.1; range, 1–7; Cronbach's alpha, 0.29). Summary knowledge scores did not differ by IUD, COC or male condom use in the unadjusted (p values of .09–.10) and adjusted (p values of .08–.10) analyses (Table 2). Of the potential confounders (age, residence, education and income), only higher income was associated with summary knowledge score (adjusted *β*, 0.43; p < .01).

**Table 2:** Association between contraceptive method use and contraceptive knowledge questions among sexually active women not desiring pregnancy in Hanoi, Vietnam, in 2017–2018

	IUC		COC		Male condoms	
	*β*	p value	*β*	p value	*β*	p value
Summary knowledge score						
Unadjusted analysis	0.21	.09	− 0.21	.10	Ref	–
Adjusted analysis^a^	0.23	.10	− 0.24	.08	Ref	–
	OR	(95% CI)	OR	(95% CI)	OR	(95% CI)
Knew that consistent condom use was best response option for preventing pregnancy						
Unadjusted analysis	0.4	(0.2–0.8)	0.3	(0.2–0.6)	Ref	–
Adjusted analysis^a^	0.5	(0.2–1.0)	0.3	(0.1–0.6)	Ref	–
Knew that male and female condoms prevent infections						
Unadjusted analysis	0.3	(0.1–1.6)	0.2	(0.0–1.2)	Ref	–
Adjusted analysis^a^	0.4	(0.1–2.3)	0.3	(0.1–1.6)	Ref	–
Identified IUD as only response option that is reversible						
Unadjusted analysis	1.2	(0.5–2.5)	1.0	(0.5–2.0)	Ref	–
Adjusted analysis^a^	1.0	(0.4–2.3)	0.9	(0.4–2.1)	Ref	–
Identified contraceptive methods that are not easily noticed by partner						
Unadjusted analysis	1.1	(0.5–2.3)	0.8	(0.4–1.6)	Ref	–
Adjusted analysis^a^	1.1	(0.5–2.6)	0.8	(0.4–1.7)	Ref	–
Identified IUD as most effective contraceptive method of response options						
Unadjusted analysis	4.7	(2.8–7.7)	0.6	(0.3–1.1)	Ref	–
Adjusted analysis^a^	4.8	(2.7–8.3)	0.5	(0.3–1.1)	Ref	–
Knew that all statements were true for IUD						
Unadjusted analysis	0.9	(0.5–1.7)	1.0	(0.6–1.9)	Ref	–
Adjusted analysis^a^	0.9	(0.5–1.7)	1.1	(0.6–2.0)	Ref	–
Knew that none of the methods cause infertility						
Unadjusted analysis	1.0	(0.6–1.6)	1.4	(0.9, 2.2)	Ref	–
Adjusted analysis^a^	1.0	(0.6–1.6)	1.5	(0.9–2.4)	Ref	–

a Adjusted for age, residence, education and income.

When we assessed the seven knowledge questions individually, only two questions were associated with contraceptive method use (Table 2). Compared to condom users, IUD and COC users had lower odds of correctly identifying consistent condom use as better for pregnancy prevention from a list of practices (i.e., using two condoms for every sex act, douching showering or bathing immediately after sex, “pulling out” before ejaculation or all equally effective). The adjusted odds ratio [aOR] for IUD and COC users was 0.5 (95% confidence interval [CI], 0.2–1.0) and 0.3 (95% CI, 0.1–0.6), respectively.

In contrast, higher proportions of IUD users (57.5%) than COC users (15.1%) and condom users (22.2%) correctly identified the IUD as the most effective contraceptive method among the possible response options (i.e., pill, male condom, withdrawal or all equally effective). Higher proportions of condom users (49.7%) mistakenly identified condoms as the most effective of the listed methods compared to IUD (20.3%) and COC users (23.0%). IUD users had an adjusted odds of answering this question correctly 4.8 times (95% CI, 2.7–8.3) that of condom users (Table 2).

## 4 Discussion

In this study of adult, reproductive-age, sexually active women attending a public hospital in Hanoi, Vietnam, for routine care, contraceptive knowledge scores did not appear to differ by type of contraceptive method currently used. The low Cronbach's alpha (0.29) for the set of seven questions that comprised the knowledge score, though, suggests that they were not measuring a single construct. Contraceptive knowledge can cover many different domains, and evaluating these separately could be important for understanding the role of knowledge and designing future counseling interventions.

We assessed the following components of contraceptive knowledge: relative effectiveness of methods, reversibility, ability to use covertly, contraindications and incorrect side effects. However, only one component, relative effectiveness of methods, was found to differ by type of method used. Condom users had higher odds of correctly identifying consistent condom use as the most effective practice from a list of possible practices, while IUD users had higher odds of correctly identifying the IUD as being more effective than the contraceptive pill, male condom and withdrawal. Higher proportions of condom users mistakenly identified male condoms instead as the most effective of these methods. Collectively, these findings suggest that users might believe that their chosen method is superior in terms of effectiveness to other methods.

In previous research, women have identified contraceptive effectiveness as one of the most important attributes for selecting a contraceptive method [3], [13], [14], [15]. Furthermore, an analysis of participants in the Contraceptive CHOICE Project highlighted the importance of knowledge of contraceptive effectiveness [16]. Those who chose to use the IUD, implant or injectable contraception had higher odds of accurately identifying the typical-use failure rates of their chosen method compared with those who opted to use the pill, patch or ring. While this analysis did not evaluate the importance of other types of knowledge, it is consistent with our findings that those using the IUD appeared to have better knowledge of the method's superior effectiveness. Two analyses of Demographic Health Survey data in Ghana and Malawi demonstrated an association between women's knowledge of their ovulatory cycles and contraception use; however, neither study evaluated the role of other types of knowledge [17], [18].

We found overall high levels of misinformation about contraception. Specifically, most women failed to identify the IUD as having higher contraceptive effectiveness relative to other methods. Furthermore, many women mistakenly believed that IUD use is inappropriate for various subgroups of women and that use of the contraceptive pill, shot or implant can cause infertility. This finding is consistent with previous literature documenting poor understanding of contraception [1], [2], [3], [4], [5], [6], [7].

Study strengths include the use of questions on a range of factors related to contraceptive knowledge. Furthermore, the study was observational in nature (i.e., without an educational study intervention) and did not include study materials (e.g., consent forms) with contraception-related information that could have influenced the findings. The relatively homogeneous sample of women — with few women declining participation — also reduces the likelihood that any differences by contraceptive method used were related to other unmeasured confounders. However, the convenience study sample also limits the generalizability of the findings to other populations, even in Vietnam. For example, the sample consisted almost exclusively of married women of Kinh ethnicity; study findings might not hold to unmarried women of other ethnicities. Furthermore, the cross-sectional study design prevents any conclusions about temporality; the act of using the IUD could have provided women more accurate information about method effectiveness during the insertion procedure or motivated them to subsequently learn, and retain, more information about its effectiveness. Finally, while the questions covered a variety of knowledge domains, they might have failed to include other influential types of knowledge.

Contraceptive counseling generally occurs somewhere along a spectrum between two extremes [19]. On one end, a directive approach is valued, in which the provider is viewed as the authority and the goal is to convince women to use a highly effective method to maximize their likelihood of avoiding unintended pregnancies. On the other end, patient autonomy is prioritized with the emphasis on ensuring that women make their own choice about contraception based on their own values, goals and beliefs. In the United States, the tension between these extremes occurs within the context of a long history of discrimination against the reproductive lives of women of color, including an initial overlap between advocates of population control and those promoting eugenics [20]. Further complicating the issue is a lack of strong evidence as to the best methods for counseling and the elements that should be covered [21], [22].

More recently, Stanback and colleagues have argued that a rights-based counseling approach requires that women understand the effectiveness of the methods to be able to make an informed choice [23]. The present study supports this argument, in that the study findings suggest that knowledge of contraceptive effectiveness could be important in decision-making regarding using a highly effective method and should be a focus of clinical counseling. In contrast, the other types of contraceptive-related knowledge that were assessed did not appear to differ by method use. Future research should take the next step of evaluating whether successfully improving women's knowledge of contraceptive effectiveness leads to the initiation and continuance of an effective method while, at the same time, ensuring that the act of choosing a highly effective method reflects the woman's true desires.

The following are the supplementary data related to this article.

Appendix Table Correct responses to contraceptive knowledge questions, by contraceptive method use Appendix Table

## Declarations of interest

None.
