# Supplementary material for: Knowledge of contraceptive effectiveness and method use among women in Hanoi, Vietnam
Source: Contracept X. 2019 Jul 11;1:100009. doi: 10.1016/j.conx.2019.100009 (PMC7252424; doi:10.1016/j.conx.2019.100009)
Supplement: Appendix Table — Correct responses to contraceptive knowledge questions, by contraceptive method use [file mmc1.docx]

| Appendix Table. Correct responses to contraceptive knowledge questions, by contraceptive method use | | | | | | |
| --- | --- | --- | --- | --- | --- | --- |
|  | IUD  (n=128) | | COC  (n=126) | | Male condoms (n=167) | |
| Questions and possible responses^*^ | No. | (%) | No. | (%) | No. | (%) |
| Which of these is best for avoiding pregnancy? |  |  |  |  |  |  |
| ***Using a condom every time you have sex*** | **100** | **(78.1)** | **93** | **(73.8)** | **150** | **(89.8)** |
| *Using two condoms every time you have sex* | 11 | (8.6) | 8 | (6.3) | 10 | (6.0) |
| *Douching, showering, or bathing immediately after sex* | 1 | (0.8) | 4 | (3.2) | 1 | (0.6) |
| *“Pulling out” before ejaculation* | 12 | (9.4) | 10 | (7.9) | 5 | (3.0) |
| *They are all equally effective* | 2 | (1.6) | 8 | (6.4) | 1 | (0.6) |
| *Don’t know or decline* | 2 | (1.6) | 3 | (2.4) | 0 | (0.0) |
| Which is the only birth control method that helps prevent infections? |  |  |  |  |  |  |
| *The birth control pill* | 1 | (0.8) | 2 | (1.6) | 0 | (0.0) |
| ***Male and female condoms*** | **123** | **(96.1)** | **120** | **(95.2)** | **165** | **(98.8)** |
| *Norplant (contraceptive implant)* | 0 | (0.0) | 2 | (1.6) | 0 | (0.0) |
| *IUD* | 1 | (0.8) | 1 | (0.8) | 0 | (0.0) |
| *Don’t know or decline* | 3 | (2.3) | 1 | (0.8) | 2 | (1.2) |
| Which of the following birth control methods may be reversed if you decide you want to become pregnant? |  |  |  |  |  |  |
| *Tubal ligation* | 1 | (0.8) | 0 | (0.0) | 2 | (1.2) |
| *Vasectomy* | 1 | (0.8) | 1 | (0.8) | 0 | (0.0) |
| ***IUD*** | **116** | **(90.6)** | **112** | **(88.9)** | **149** | **(89.2)** |
| *None of the above* | 6 | (4.7) | 5 | (4.0) | 9 | (5.4) |
| *Don’t know or decline* | 4 | (3.1) | 8 | (6.3) | 7 | (4.2) |
| Which birth control method is not easily noticed by a partner† |  |  |  |  |  |  |
| ***IUD*** | **8** | **(6.3)** | **6** | **(4.8)** | **5** | **(3.0)** |
| ***Depo-Provera (“the shot”)*** | **65** | **(50.8)** | **53** | **(42.1)** | **59** | **(35.3)** |
| *Norplant (contraceptive implant)* | 9 | (7.0) | 11 | (8.7) | 14 | (8.4) |
| ***They all aren’t easily noticed*** | **42** | **(32.8)** | **50** | **(39.7)** | **85** | **(50.9)** |
| *Don’t know or decline* | 4 | (3.1) | 6 | (4.8) | 4 | (2.4) |
| Which method of birth control is the best at preventing pregnancy? |  |  |  |  |  |  |
| ***IUD*** | **73** | **(57.5)** | **19** | **(15.1)** | **37** | **(22.2)** |
| *The pill* | 3 | (2.3) | 46 | (36.5) | 14 | (8.4) |
| *Male condom* | 26 | (20.3) | 29 | (23.0) | 83 | (49.7) |
| *Withdrawal (“pull-out method”)* | 3 | (2.3) | 3 | (2.4) | 3 | (1.8) |
| *They are all equally effective* | 20 | (15.6) | 26 | (20.6) | 29 | (17.4) |
| *Don’t know or decline* | 3 | (1.6) | 3 | (2.4) | 1 | (0.6) |
| Which statement is true about IUDs? |  |  |  |  |  |  |
| *Women of all ages may get an IUD* | 12 | (9.4) | 16 | (12.7) | 16 | (9.6) |
| *Women who have never had a baby may get an IUD* | 2 | (1.6) | 1 | (0.8) | 2 | (1.2) |
| *Women can have an IUD put in right after having a baby or having an abortion* | 65 | (50.8) | 49 | (38.9) | 73 | (43.7) |
| ***They are all true*** | **23** | **(18.0)** | **25** | **(19.8)** | **32** | **(19.2)** |
| *Don’t know or decline* | 26 | (20.3) | 35 | (27.8) | 44 | (26.3) |
| Which method of birth control can cause infertility? |  |  |  |  |  |  |
| *The pill* | 19 | (14.8) | 12 | (9.5) | 26 | (15.6) |
| *Depo-Provera (“the shot”)* | 4 | (3.1) | 0 | (0.0) | 4 | (2.4) |
| *Norplant (contraceptive implant)* | 2 | (1.6) | 1 | (0.8) | 3 | (1.8) |
| ***None of the above*** | **72** | **(56.3)** | **81** | **(64.3)** | **94** | **(56.3)** |
| *Don’t know or decline* | 31 | (24.2) | 32 | (25.4) | 40 | (24.0) |
| IUD = intrauterine device  ^*^Bold text indicates the correct response  †The intended answer was “they aren’t all easily noticed,” but because the implant could be detectable, especially in women with low body mass index, “IUD” and “Depo-Provera” also were scored as correct. | | | | | | |
